# A multimodal material route planning problem considering key processes at work zones

**DOI:** 10.1371/journal.pone.0300036

**Published:** 2024-06-06

**Authors:** Youmiao Wang, Rui Song, Ziqi Zhao, Rixin Zhao, Zheming Zhang

**Affiliations:** Key Laboratory of Transport Industry of Big Data Application Technologies for Comprehensive Transport, Beijing Jiaotong University, Beijing, China; Al Mansour University College-Baghdad-Iraq, IRAQ

## Abstract

With the continuous development of large-scale engineering projects such as construction projects, relief support, and large-scale relocation in various countries, engineering logistics has attracted much attention. This paper addresses a multimodal material route planning problem (MMRPP), which considers the transportation of engineering material from suppliers to the work zones using multiple transport modes. Due to the overall relevance and technical complexity of engineering logistics, we introduce the key processes at work zones to generate a transport solution, which is more realistic for various real-life applications. We propose a multi-objective multimodal transport route planning model that minimizes the total transport cost and the total transport time. The model by using the *ε* − constraint method that transforms the objective function of minimizing total transportation cost into a constraint, resulting in obtaining pareto optimal solutions. This method makes up for the lack of existing research on the combination of both engineering logistics and multimodal transportation, after which the feasibility of the model and algorithm is verified by examples. The results show that the model solution with the introduction of the key processes at work zones produces more time-efficient and less time-consuming route planning results, and that the results obtained using the *ε* − constraint method are more reliable than the traditional methods for solving multi-objective planning problems and are more in line with the decision maker’s needs.

## 1 Introduction

With the orderly advancement of the new technological revolution and the gradual emergence of new technical means, constructing new infrastructures has gradually become a key area of infrastructure layout. The Global Infrastructure Outlook (GIO) report states that to support the needs of global economic growth, the cost of infrastructure will reach US$94 trillion in 2040. Furthermore, the UK government’s National Infrastructure Strategy outlines plans to implement various infrastructure-related policies in the coming years while increasing investments. With the advent of the post-epidemic era, global infrastructure construction has started to recover, leading to the gradual launch of numerous large-scale engineering projects.

Engineering logistics is a type of logistics service that revolves around engineering projects. It encompasses the planning, organization, coordination, and control of the material transportation process, from the extraction of raw materials to the completion of the project [1]. Logistics links, a wide range of services, involving a large scale, and the operation of the technical level of high requirements is an essential feature of engineering logistics. The four main modes of transport used in engineering logistics are rail, road, waterway and air. Usually, railway and waterway are preferred for long-distance transport, while road is used for close distance, and air transport is considered for time urgency. This is determined by the transport demand and transport conditions of large-scale engineering projects. Large-scale engineering projects involve a great demand for engineering materials, and suppliers are scattered throughout the more dispersed. According to the progress of the project accurately planning the transport solution of engineering materials is usually very important. The route planning problem is one of the most important and widely researched problems in the process of engineering logistics cargo transport [2]. In the traditional route planning problem model, there is no priority between demands [3]. However, in practical situations, different demands cannot be considered equally important.

In this paper, we study the multimodal material route planning problem (MMRPP) since it serves as an indispensable part to generate the transport solutions of the engineering logistics. Therefore, the MMRPP problem needs to be solved effectively to guarantee the accurate distribution of engineering materials. As we all know, in the process of construction of large engineering projects, the project construction work will be carried out by dividing the work area, and each work area will carry out the project construction work together, so each work area has different demands for engineering materials. If the distribution of project materials is completed too early, it will lead to excessive stacking of materials in the work zones, or even nowhere to stack; while delayed distribution will lead to no materials in the work zones to carry out the project construction work, delaying the progress of project completion. In recent years, scholars have conducted various research on the transport route planning of engineering materials from different perspectives. Among them, the transport route planning method that takes economy and timeliness as the goal and considers a single transport mode has been widely noticed and recognized. At the same time, there are also a large number of studies on multimodal transportation route planning, scholars from the solution of practical problems, for different transportation objects, such as hazardous materials, medical wastes, containers, etc., constructed the multimodal transportation route planning model, using different solution algorithms to derive the optimal route. However, in the existing research, there are fewer literatures applying multimodal transportation to the transportation route planning of engineering materials. Some of the literature on engineering materials transportation route planning adopts multimodal transportation, but there is no discussion on the characteristics and demand of engineering materials transportation in large-scale engineering projects, and very few of the literature considers the priority of the demand of engineering materials in different work zones.

The main contribution of this paper is to introduce multimodal transportation into the field of engineering logistics, and put forward the concept of key processes in the work zones according to the characteristics of large-scale engineering project and engineering materials transportation to ensure the timely supply of materials. We first construct a multi-objective route planning model considering multimodal transport and enhance the model for the consideration of work zone demand priority by adding work zone key process variables. Then, we involve an *ε* − constraint method to compute the route planning results of the proposed model. Finally, we verify the effectiveness of the proposed algorithm by comparing its performance with that of existing algorithms for solving multi-objective planning problems in real cases.

The remainder of the paper is organized as follows: Section 2 reviews the relevant literature. In Section 3, we introduce the concept of the key processes at work zones and construct a multi-objective route planning model. In Section 4, we use a *ε* − constraint method to solve the model in Section 3. In Section 5, we conduct a case study and discuss parameter variations. Section 6 gives conclusions and suggestions for future work.

## 2 Literature review

The research on engineering logistics was initially proposed by American scholars, who primarily emphasized the significance and application of optimizing transportation solutions in international engineering logistics. Silva et al. [4] proposed the theory of delivering construction materials to the project site on time and as needed. Guffond et al. [5] emphasized that engineering logistics should be tailored to the specific needs of engineering goods, utilizing all available resources to transport materials to the construction site smoothly, punctually, and cost-effectively. Sobotka et al. [6] highlighted that effective logistics management of project materials is a critical factor influencing the overall construction schedule. Andrew et al. [7] conducted a brief analysis of the factors to consider in developing a sound transport solution, such as the impact of appropriate transport methods, suitable tools, and streamlining the process on reducing total transport costs. Yang et al. [8] explored the characteristics of international project logistics and developed an optimization model for transport solutions based on decision network planning techniques and uncertainty theory. Stochastic programming theory was employed to solve the model. Osawaru et al. [9] conducted a study on material supply planning for construction projects and utilized random sampling techniques. The study concluded that well-planned construction material procurement effectively prevents project delays caused by untimely ordering and delivery of materials. Most of the aforementioned studies aimed to minimize costs and optimize the logistics and transport solutions for engineering projects by analyzing the influencing factors, without giving specific routes for transporting engineering materials.

As engineering materials often possess characteristics such as being overweight, overlength, and overwidth, transportation becomes challenging, carrying significant operational risks. Determining the most suitable transport solutions becomes crucial to ensure the smooth and accessible transportation of materials. Constructing a model to address path selection in logistics transportation schemes for engineering projects, particularly when multiple alternative transport solutions exist between the starting point and the endpoint, becomes a vital step in ensuring the feasibility of the engineering logistics transportation plan. Zhang et al. [10] formulated a dynamic transportation planning problem based on a just-in-time (JIT) strategy and applied a particle swarm optimization (PSO) algorithm to solve a dynamic transportation planning problem for PCSC. Song et al. [11] conducted a study on cargo routing optimization and empty container relocation problems within a shipping network comprising multiple service routes, deployed vessels, and scheduled voyages. They proposed two approaches: an integer planning method based on a two-stage shortest path and an integer planning method based on a two-stage heuristic rule to address these challenges. Niu et al. [12] developed an integrated MiC logistics planning and visualization platform based on Building Information Modelling (BIM), Geographic Information System (GIS) and Vehicle Routing Problem (VRP) algorithm integration. This platform aims to determine the most optimal logistics solution for trailer routes, ensuring the timely installation of MiC projects in Hong Kong within the designated time window. Abdzadeh et al. [13] proposed a transport path model based on MDOVRP that takes into account realistic conditions such as multi-project environments, incremental discounts and quality checks. To solve the model efficiently, they developed a method based on a taboo search algorithm. Xiong et al. [14] proposed a cold chain logistics distribution path optimization scheme based on an improved ant colony optimization algorithm (IACO). Zeng et al. [15] transformed the influencing factors in the transportation process into edge weights, utilizing the characteristics of building material logistics and complex network theory. The average shortest path was then calculated to guide the selection of transport routes for building material logistics. Almashaqbeh et al. [16] constructed a model with the objective of minimizing the total transport and storage costs of prefabricated modules in modular building projects. Liu et al. [17] applied an immune algorithm, a q-value method and an improved ant colony algorithm to the model to solve the route planning problem of medical waste transportation. Wang et al. [18] proposed a theoretical two-stage decision-making method (TDM) that combines the path optimization method and multi-criteria decision-making (MCDM) method to solve the bulk material transportation problem in an innovative manner. Men et al. [19] proposed a multi-objective robust VRPTW (MO-RVRPTW) model for the transportation of dangerous goods, considering both multi-objective optimization and uncertainty. This model aims to optimize the number of vehicles while addressing uncertain transportation risks. Ma et al. [20] constructed a risk-balanced multi-distribution center dangerous goods vehicle robust scheduling problem, incorporating four objectives: risk minimization, cost minimization, risk equilibrium value minimization, and duration minimization.

In recent years, with the rapid growth of international trade, multimodal transport has gained increasing popularity in solving route planning problems. Perboli et al. [21] proposed a simulation-optimization framework for constructing examples and evaluating operational settings to address the application of multimodal transport in urban logistics last-mile distribution. Filippova et al. [22] improved the reliability and safety of intermodal transport in the Arctic by establishing a transport and logistics center for multimodal transport management. Turbaningsih et al. [23] employed a mixed-methods approach, utilizing qualitative research and observational transport analysis, to study the main criteria for proposing improved strategies in multimodal transport of heavy cargo. Xiong et al. [24] focused on the problem of multimodal transport path optimization with time windows. They established a mathematical model with two optimal objectives, multiple available modes of transport and different demand delivery times. To address this, they designed a two-layer multi-objective genetic algorithm to solve the problem. Hao et al. [25] proposed an optimization model based on dynamic programming to obtain the optimal combination strategy for organizing various transportation modes in the container intermodal transport system. This model considered factors such as time, cost, and cargo transportation quality. Luo et al. [26] considered the need for route or node reconstruction and proposed an OHC path planning reconfiguration model. This model aims to simultaneously determine transport routes, transport modes, and the routes or nodes that require reconstruction to minimize the total cost. Li et al. [27] established a multi-objective fuzzy nonlinear programming model considering mixed-time window constraints by taking cost, time, and carbon emission as optimization objectives, and a cooperative game theory-based multi-objective optimization method is proposed. Zhu et al. [28] considered the uncertainty in highway transport speed and transshipment time in the actual transport process, established a multi-objective path-decision models of multimodal transport under different carbon policies, used the law of large numbers to estimate the expected value of nonlinear uncertainty, and used the K-shortest path algorithm and the non-dominated sorting algorithm (NSGA-II) to solve the model. Kaewfak et al. [29] developed a decision support model using an analytic hierarchy process (AHP) and zero-one goal programing (ZOGP) to determine an optimal multimodal transportation route. Fathollahi-Fard et al. [30] constructed a bi-level model for a home healthcare supply chain (HHCSC) that considers outsourcing demand and considers two levels (i.e., leaders (nurses) and followers (patients)) for the decision variables, and a bi-level meta-heuristic approach was adopted to solve the model; after that, it was further optimized with the objectives of total cost, environmental pollution, and total unemployment time, a sustainable home healthcare logistics model was developed by incorporating economic, environmental sustainability, and social factors into the model, and a combination of the epsilon constraint method and the Lagrangian relaxation theory was proposed [31].

Table 1 discussed the multimodal transportation route planning problem from multiple perspectives, and a further summary of the literature in terms of transportation method, objects, result form and solution methodology reveals that the existing research on the multimodal transportation route planning problem involves a lot kinds of transportation objects, the solution methodology are mostly heuristic algorithms, and the output results are mostly the optimal paths. But there is a lack of specific studies for the field of engineering logistics, and there is no investigation of the characteristics of the large-scale engineering project influence on the transportation process of engineering materials, as well as combining the key process characteristics of the engineering project duration of engineering materials for path planning. Therefore, this study aims to establish a multi-objective route planning model and the related algorithm for engineering materials considering multimodal transport.

**Table 1:** Summary of the relevant studies and present research.

Reference	Transportation Method	Transportation Objects	Objective Function	Solution Methodology	Result Form	Engineering Logistics
Zhang H, et al.[10]	Highway	Prefabricated component	Minimizing the transportation costs	The particle swarm optimization	Overall logistics planning considering supplier selection, initial transportation plan, site layout plan and transportation plan adjustment	√
Dong-Ping Song, et al.[11]	Shipping	Container	Minimizing the total relevant costs	A two-stage shortest-path based integer programming method、a two-stage heuristic-rules based integer programming method	Shipping service routes	-
Niu S, et al.[12]	Road Transportation	Modular integrated structural building materials	Meet the installation time window	The ABC algorithm	Optimal logistics solution consisting of three routes	√
Xiong H.[14]	Road Transportation	Cold Chain	Minimizing the total cost	Ant Colony Optimization Algorithm	The optimal logistics distribution path	-
Zeng R, et al.[15]	Multimodal transportation	Construction logistics	Minimizing transport cost, distance, and risk	The complex network theory	The optimal path	√
Liu Z, et al.[17]	Road Transportation	Medical Waste	Minimizing the transportation path	A Hybrid Optimization Algorithm Based on the Immune–Ant Colony Algorithm	Path planning results	
Men J, et al.[19]	Road Transportation	Hazardous materials	Minimizing the number of vehicles and the transportation risk	Hybrid evolutionary algorithm	A set of robust non-dominated solutions	-
Ma C, et al.[20]	Road Transportation	Hazardous materials	Minimizing the transportation cost、 the difference value of transportation risk and the duration of the transportation task	Hybrid particle swarm optimization and genetic algorithm	The specific driving path	-
Hao C, et al.[25]	Multimodal transportation	Container	Minimizing the generalized transport cost	The breadth—first search algorithm and the dynamic programming algorithm	The best route	-
Luo Y, et al.[26]	Multimodal transportation	Oversize and heavyweight cargo	Minimizing the total cost	Improved KSP algorithms	Transportation schemes	√
Li L, et al.[27]	Multimodal transportation	-	Minimizing transportation cost, transportation time, and transportation carbon emission	A cooperative game theory-based multi-objective optimization method	Transportation Path	-
Zhu, et al.[28]	Multimodal transportation	Phosphate	Minimizing the total carbon emissions and transportation time	the K-shortest path algorithm and NSGA-II	Transportation Path	-
Kaewfak, et al.[29]	Multimodal transportation	Coal manufacturing	-	AHP and ZOGP	The optimal route	-
A.M. Fathollahi-Fard, et al. [30]	Road Transportation	Home Health Care Supply Chain	Minimizing the cost of selecting pharmacies and the total cost of visiting the patients	A solution procedure combining a heuristic and exact method	The optimal route	-
A.M. Fathollahi-Fard, et al. [31]	Road Transportation	Healthcare logistics	Minimizing the total cost, environmental pollution, and total unemployment time	A combination of the epsilon constraint method and the Lagrangian relaxation theory	The optimal route	-

## 3 Problem description and mathematical formulation

In this section, the problem statement is introduced. Next, the main assumptions for the proposed problem and symbol descriptions are illustrated. Finally, a multi-objective route optimization model considering the key processes of work zones is developed.

### 3.1 Problem description

**Fig 1:**
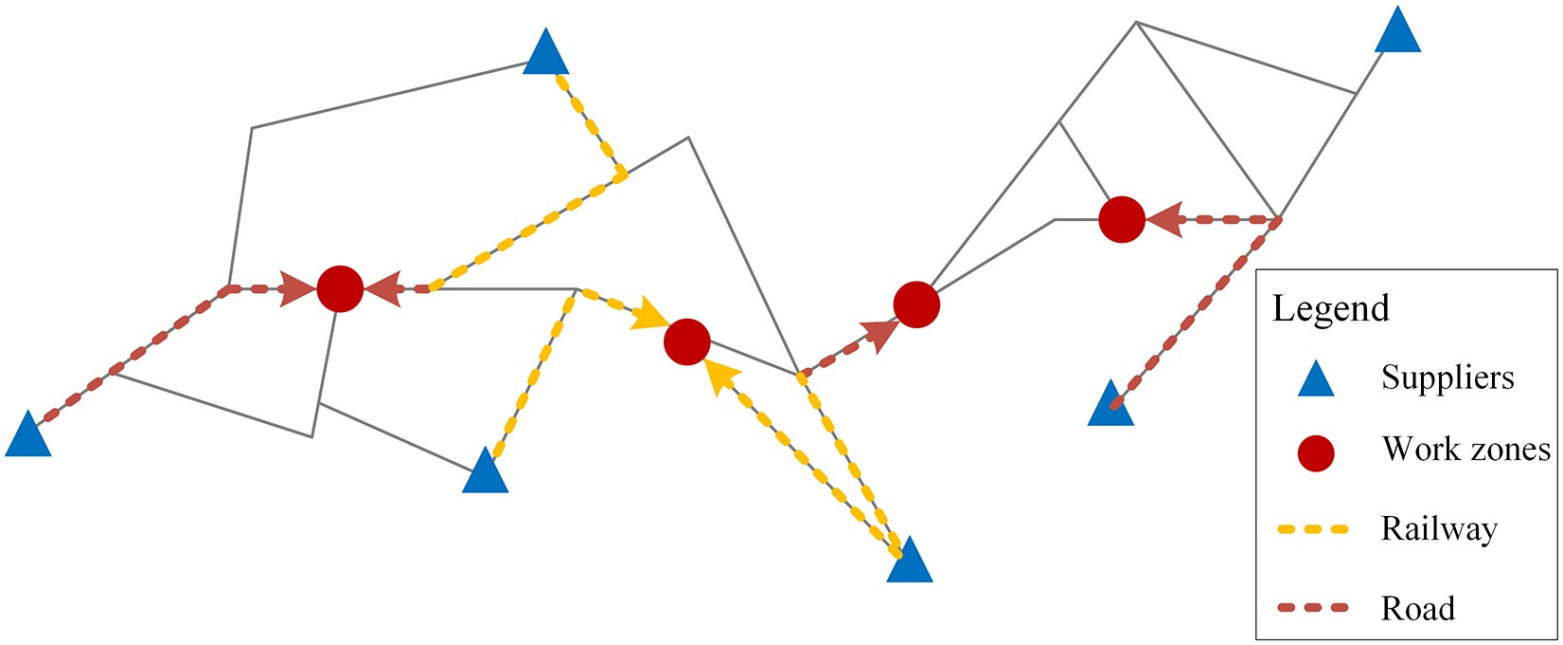
Sketch of the transport of materials for large engineering projects.

In the construction of large-scale engineering projects, the supply of engineering materials is organized into cycles. Each work area submits material demands to the supplier based on the progress of their respective projects, typically at regular time intervals, such as weekly or biweekly. The supplier then transports the requested materials to the work areas accordingly. The urgency of material demands varies depending on the importance of different work zones during the project construction process, and this urgency is primarily determined by the work processes of each work zone. Additionally, due to the characteristics of engineering construction materials supply, the transportation process usually involves multimodal transport and mode conversion. Fig 1 briefly illustrates the process of transporting materials for a large project.

In this paper, we propose a multi-objective route optimization model considering the key processes of work zones. The main objective is to satisfy the material demands of multiple work zones by constructing a multi-objective route optimization model that considers both fairness (prioritizing work zones with earlier work processes) and efficiency (minimizing transportation costs). This model considers the work processes of different work zones for the same type of materials and determines the material transport solutions under the specified constraints.

To simplify the model, the following are assumed [26, 32]:

1. The demand for supplies in each work zone is known and the supplier’s stock of supplies can meet the demand for supplies in all work zones.
2. The fleet of vehicles provided by the supplier has sufficient capacity to deliver all the supplies to the work zones.
3. The number and geographical locations of the supplier and the work zones are known.
4. There is no movement of engineering materials between work zones.
5. The materials of a certain category in a work zone as a whole cannot be divided, which means that goods cannot be transmitted into more than one mode simultaneously. The transfer of goods can only occur at designated nodes and not during transit.
6. Goods can undergo mode of transportation transfer at each node, but each node can only be visited at most once. This means that goods between two nodes can only select one mode of transportation for transporting goods.
7. Each OD (origin-destination) traffic stream has no change in traffic flow when passing through each node.
8. During the decision-making cycle, the capacity of each node and line in the network topology of the work area is predetermined, and the entire network structure is stable in terms of scale and structure.

**Definition of set**:

*D* = {*d* | *d* = 1, 2, …, *d*}—node set of work zones in the transportation network.

*S* = {*s* | *s* = 1, 2, …, *s*}—node set of suppliers in the transportation network.

*I* = {*i* | *i* = 1, 2, …, *i*}—collection of nodes in the transportation network other than work zones and suppliers.

*N* = *D* ∪ *S* ∪ *I*—collection of all nodes in the transportation network.

Γ—collection of road sections in the transportation network, (*i*, *j*) ∈ Γ, *i* ∈ *N*, *j* ∈ *N*.

*K* = {*k* | *k* = 1, 2, …, *k*}—collection of transport modes.

**Parameters**:

$C_{ij}^{k}$—unit cost of the transport mode *k* on the road section (*i*, *j*), *k* ∈ *K*, (*i*, *j*) ∈ Γ.

$C_{i}^{kl}$—transit cost when the transport mode changed from *k* to *l* on the node *i*, *k*, *l* ∈ *K*, *i* ∈ *I*.

$L_{ij}^{k}$—distance of the transport mode *k* on the road section (*i*, *j*), *k* ∈ *K*, (*i*, *j*) ∈ Γ.

$P_{ij}^{k}$—capacity of the transport mode *k* on the road section (*i*, *j*), *k* ∈ *K*, (*i*, *j*) ∈ Γ.

$P_{i}^{k}$—capacity of the transport mode *k* on the node *i*, *k* ∈ *K*, *i* ∈ *N*.

*H*_*s*_—material inventory of the supplier *s*, *s* ∈ *S*.

*B*_*d*_—Material requirement of the work zone *d*, *d* ∈ *D*.

*Num*—Number of suppliers in the transport network.

*Speed*_*k*_—average speed of transport mode *k*, *k* ∈ *K*.

*T*_*d*_—maximum transport time for the work zone *d*, *d* ∈ *D*.

*X*_*d*_—key process of the work zone *d*, *d* ∈ *D*.

*CX*_*d*_—processed key process of the work zone *d*, *d* ∈ *D*.

$t_{ij}^{k}$—transport time spent by transport mode *k* on the road section (*i*, *j*), *k* ∈ *K*, (*i*, *j*) ∈ Γ,$t_{ij}^{k}=L_{ij}^{k}/Speed_{k}$.

$t_{i}^{kl}$—transit time spent by transport mode changed from *k* to *l* on the node *i*, *k*, *l* ∈ *K*, *i* ∈ *I*.

**Decision variables**:

$$x_{ij}^{kd}=\left\{\begin{array}{cc} 1, & \begin{array}{c} \text{the}\;\text{flow}\;d\;\text{passes}\;\text{through}\;\text{the}\;\text{road}ij\;\\ \text{by}\;k\text{th}\;\text{transport}\;\text{mode}\;\text{and}\;(i,j\text{)}\in I,d\in D,k\in K \end{array}\\ 0, & \text{otherwise} \end{array}\right..$$

$$y_{id}^{kl}=\left\{\begin{array}{cc} 1, & \begin{array}{c} \text{the}\;\text{flow}\;d\;\text{changes}\;\text{the}\;\text{transport}\;\text{mode}\;\\ \text{from}\;k\;\text{to}\;l\;\text{and}\;d\in D,k\in K,l\in K \end{array}\\ 0, & \text{otherwise} \end{array}\right..$$

### 3.2 Mathematical formulation

In this model, the concept of the key processes of the work zones *X*_*d*_ is introduced, *X*_*d*_ = {*X*_*d*_ | *X*_*d*_ = 1, 2, ⋯, *Num*}, *d* ∈ *D*. Typically, in large-scale engineering projects, different work zones are divided based on geographical locations or other dimensions according to the construction situation. These work zones are simultaneously carried out throughout the project but still have significant differences in importance. The key processes at work zones indicate the order of the work zones in the entire project, which means that a smaller value of *X*_*d*_ indicates a higher priority in the construction sequence for that work zone. Therefore, when planning the route of material demands of multiple work zones of a project, it is necessary to consider the key processes at work zones to ensure sufficient support for materials required in work zones with higher priority construction sequences. The key processes at work zones *X*_*d*_ is a natural number, and its numerical value indicates the priority construction sequence of the work zone. The model constructed in this paper takes the minimization of cost and time as the optimization objective. To better align with practical considerations, the reciprocal of the key processes at work zones *X*_*d*_ is taken and weighted into the corresponding objective functions, resulting in *CX*_*d*_.

The multi-objective route optimization model is established as follows:

$$\text{Min}Z_{1}=\sum_{d\in D}\sum_{k\in K}\sum_{(i,j)\in N}CX_{d}\cdot x_{ij}^{kd}\cdot t_{ij}^{k}+\sum_{d\in D}\sum_{k,l\in K}\sum_{(i,j)\in N}CX_{d}\cdot y_{id}^{kl}\cdot t_{i}^{kl}$$ (1)

$$\text{Min}Z_{2}=\sum_{d\in D}CX_{d}\cdot B_{d}\cdot (\sum_{k\in K}\sum_{(i,j)\in N}x_{ij}^{kd}\cdot C_{ij}^{k}\cdot L_{ij}^{k}+\sum_{d\in D}\sum_{k,l\in K}\sum_{(i,j)\in N}y_{id}^{kl}\cdot C_{i}^{kl})$$ (2)

$$\sum_{k,l\in K}y_{id}^{kl}\leq 1,\forall i\in N,\forall d\in D$$ (3)

$$\sum_{k\in K}x_{ij}^{kd}\leq 1,\forall (i,j)\in \Gamma ,\forall d\in D$$ (4)

$$\sum_{(j,i)\in \Gamma }x_{ji}^{kd}+\sum_{(i,j)\in \Gamma }x_{ij}^{ld}\leq 2y_{id}^{kl},\forall i\in N,d\in D,k\in K,l\in K$$ (5)

$$\sum_{k\in K}\sum_{i,j\in \Gamma }x_{ij}^{kd}\cdot t_{i,j}^{k}+\sum_{k,l\in K}\sum_{i\in N}y_{id}^{kl}\cdot t_{i}^{kl}\leq T_{d},\forall d\in D$$ (6)

$$\sum_{d\in D}\sum_{k\in K}\sum_{(s,j)\in \Gamma }x_{sj}^{kd}\cdot B_{d}\leq H_{s},\forall s\in S$$ (7)

$$\sum_{(i,j)\in \Gamma }\sum_{k\in K}x_{ij}^{kd}-\sum_{(j,i)\in \Gamma }\sum_{k\in K}x_{ji}^{kd}=\left\{\begin{array}{ll} 1, & i=s\\ 0, & i\neq s\;\text{and}\;i\neq d\\ -1, & i=d \end{array}\right.,\;\forall d\in D,\forall i\in N$$ (8)

$$\sum_{d\in D}x_{ij}^{kd}\cdot B_{d}\leq P_{ij}^{k},\forall (i,j)\in \Gamma ,\forall k\in K$$ (9)

$$\sum_{d\in D}y_{id}^{kl}\cdot B_{d}\leq P_{i}^{k},\forall i\in N,\forall k,l\in K$$ (10)

Where (1) and (2) is the objective function. Formula (1) minimizes the total time spent in the process of transporting materials, with the first term representing the transport time from the supplier to the work area and the second term denoting the transit time at each intermediate node where a change in the transport mode occurs. Formula (2) minimizes the total cost, where the first term is the cost of transporting materials from the supplier to the work area, and the second term represents the cost of transit operations at each transit node where a change in the transport mode occurs. Formula (3) indicates that each transport task can only undergo no more than one transport mode change at each transport node. Formula (4) suggests that for the same cargo flow, only one mode of transport is allowed between two neighboring transport nodes on its route, i.e., the cargo cannot be divided again during transport. Formula (5) ensures the continuity of transport mode transformation, when transporting between two neighboring transport nodes. A change of the transport mode can only occur at the node of the transport network when the transport task arrives at the node via transport mode *k* and leaves the node via transport mode *l*. Formula (6) is the arrival time limitation constraint, which ensures that the arrival time of all transport tasks is within the permitted time of the corresponding work zone. Formula (7) is the supplier supply capacity limitation constraint, indicating that the total transport material outflow from a supplier should be less than the inventory of that supplier. Formula (8) represents the transport network route selection flow balance constraint. Formula (9) is an arc capacity constraint that ensures that all transport flows do not exceed the transportation capacity limit for each transport mode on each arc. Formula (10) represents the node capacity constraint, ensuring that the volume of transshipment operations between multiple transport modes does not exceed the node capacity limit.

## 4 Proposed algorithm

The current methods for solving multi-objective planning problems mainly include linear weighting method, *ε* − constraint method, ideal point method and hierarchical sequence method. The linear weighting method is the most widely used method, which converts an unmanageable multi-objective problem into a traditional single-objective problem by linearly weighting the multiple objectives of the problem and representing them with a comprehensive utility function. However, the linear weighting method requires scaling each objective and developing weights for different objectives that indicate the importance that the decision maker attaches to different aspects of the problem. For the model objectives in this paper, the two scales are not uniform. Also, the decision maker is not able to formulate convincing enough weights for each objective before solving, so this method is not considered. The ideal point method is to find the optimal solution of the problem under each individual objective, and then let the objective approach the optimal value of each objective, i.e., to minimize the difference between the objective value of the solution and the optimal value as the goal. However, this method requires a uniform scale and also requires setting weights for the differences of each objective, so the ideal point method is also not considered. The hierarchical sequence method is a multiple iteration solution method in which only one objective is treated in each iteration. First of all, the method needs to rank multiple objectives according to their importance; then iteratively solve the model, each time the solution, the objective is set to the highest importance of the unsolved objectives, the solved objectives as constraints into the model, and after the end of the optimal value of the second most important objective under the premise of the optimization of the more important objectives can be found. However, the objectives in this paper do not have a clear order of importance, so the method is not suitable for the model in this paper.

In the model constructed in this paper, when the planning route selects a faster transport mode to save transport time, it may lead to an increase in transport cost. Conversely, when the transport mode with lower transport cost is selected, it may increase the transport time. In this decision-making process, judgement can be made based on the theory of Pareto optimality. Pareto optimality is not unique, but effective Pareto optimality is liberated together to form a Pareto frontier. Based on the Pareto frontier, the decision maker can select the appropriate solution according to their own needs. How to obtain the Pareto optimality and its Pareto frontier is a key problem to be solved.

The *ε* − constraint method is to solve problem by dividing the original multiple optimization objectives into main objectives and other sub-objectives and using the other sub-objectives as constraints. A large number of studies applying the *ε* − constraint method to the solution of multi-objective routing models have demonstrated the effectiveness of the *ε* − constraint method in solving such problems [33, 34]. In this paper, the *ε* − constraint method [35] is chosen to solve the Pareto optimality. The method is defined as follows:

$$\text{Min}f_{1}$$ (11)

$$s.t.f_{i}\leq \varepsilon_{i}\;\forall i=2,\ldots ,n$$ (12)

**Fig 2:**
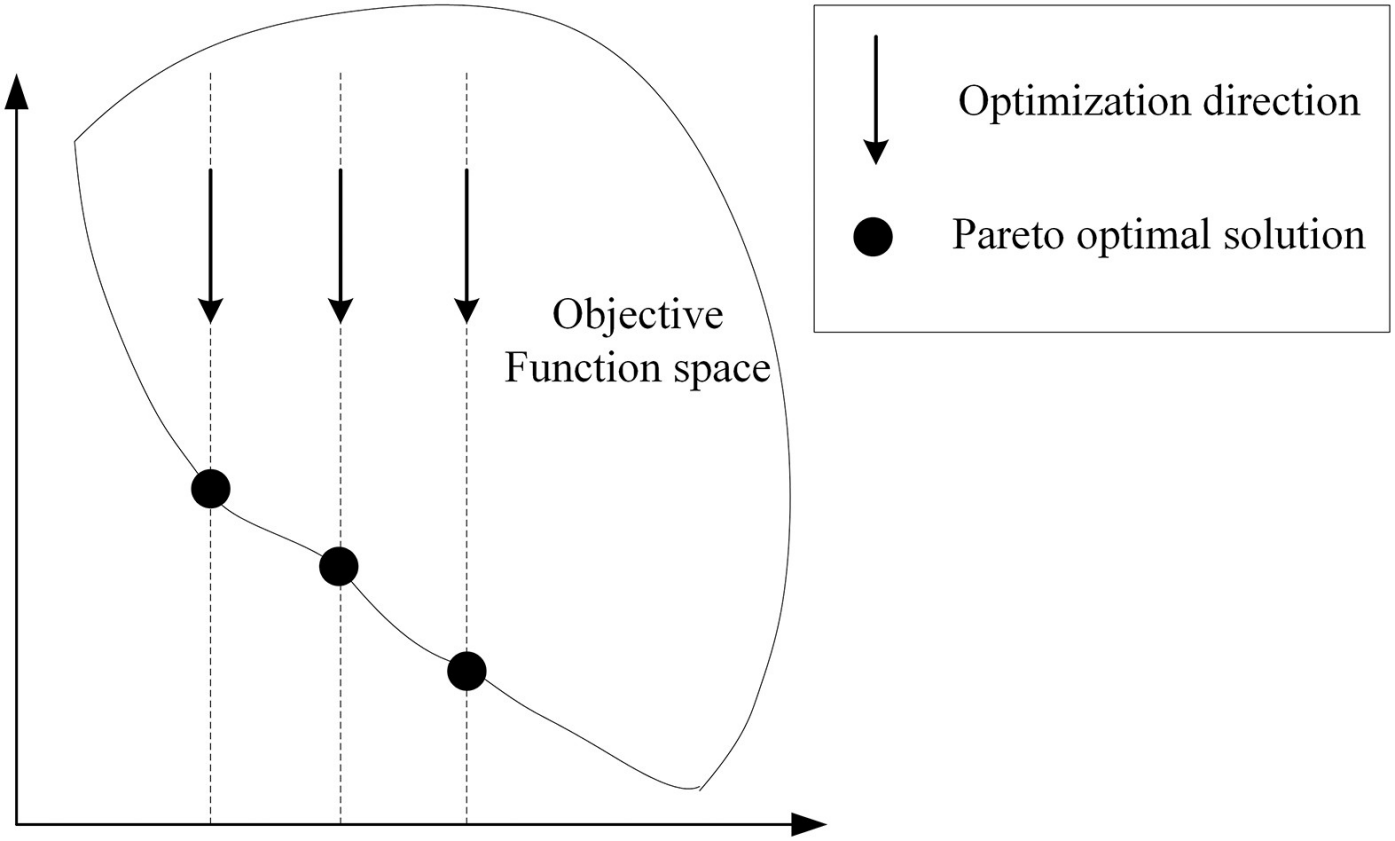
*ε* − constraint method.

Where *f*_1_, …, *f*_*n*_ is the nth objective function of the problem and *ε*_*i*_ is the upper bound of the *i*th objective. Fig 2 shows how the method works.

The *ε* − constraint method first derives the optimal value of each individual objective, then selects one objective as the main objective of the model according to the preference, and converts the other objectives into constraint considerations. Using *ε* to deflate the optimal value of other objectives, the other objectives in the solution process only need to be within the range before and after the deflation of the optimal value to satisfy the constraints. This not only ensures the optimization of the main objective, but also takes other objectives into account. When using the *ε* − constraint method to solve the model, the value of *ε* needs to be constantly adjusted to change the constraint range of other objectives to obtain a series of optimal solutions, which form the Pareto boundary. Each point on the Pareto boundary represents a Pareto optimal solution, and for each Pareto optimal solution, it is not possible to solve to obtain a solution that is better than each objective value. That is, it is not possible to change these Pareto-optimal solutions so that one objective value is better while the other objectives are not worse. Once the Pareto bounds are obtained, the results can be selected according to the decision maker’s preferences.

In this paper, the main objective chosen for optimization is the minimization of the total transport time to ensure faster delivery of construction materials to the site. We set the *ε* value within the range between the optimal value of the cost obtained by solving the model with the cost objective only and the transport time objective only. Denote the original model as *P*_0_ and the transformed model as *P*_1_. The solution process as follows:

Step1: Calculate the constraint lower bound.
  Keeping only the objective function *Z*_2_, solve the model *P*_0_ by minimizing the transport cost as the objective function and calculate the optimal solution of the transport cost,$min_{cost}^{lb}$. The lower bound of the objective function *Z*_2_ is taken as $min_{cost}^{lb}$, and the total transport time corresponding to this solution is taken as $min_{time}^{ub}$, i.e., the upper bound of the objective function *Z*_1_.
Step2: Calculate the constraint upper bound.
  Keeping only the objective function *Z*_1_, solve the model *P*_0_ by minimizing the transport time as the objective function and calculate the optimal solution of the transport time,$min_{time}^{lb}$. The lower bound of the objective function *Z*_1_ is taken as $min_{time}^{lb}$, and the total transport cost corresponding to this solution is taken as $min_{cost}^{ub}$, i.e., the upper bound of the objective function *Z*_2_.
Step3: Determine the range of the sub-objective values is $\left[min_{cost}^{lb},min_{cost}^{ub}\right]$ and convert the objective function *Z*_2_ into constraint according to the upper and lower limits:
$$\begin{array}{c} \sum_{d\in D}CX_{d}\cdot B_{d}\cdot (\sum_{k\in K}\sum_{(i,j)\in N}x_{ij}^{kd}\cdot C_{ij}^{k}\cdot L_{ij}^{k}+\sum_{d\in D}\sum_{k,l\in K}\sum_{(i,j)\in N}y_{id}^{kl}\cdot C_{i}^{kl})\leq \varepsilon_{i},\\ min_{cost}^{lb}\leq \varepsilon_{i}\leq min_{cost}^{ub} \end{array}$$ (13)
Step4: Calculate the adjustment scheme under the setting constraints and solve the model *P*_1_ to get the current optimal solution.
Step5: Update the constraint value, *ε*_*i*_ = *ε*_*i*_ + *δ*, *δ* for the step size.
Step6: Judge whether the constraint value is the minimum value of the optimization objective, if $\varepsilon_{i}\in [min_{cost}^{lb},min_{cost}^{ub}]$, then turn to step4; if $\varepsilon_{i}\notin [min_{cost}^{lb},min_{cost}^{ub}]$, then continue to step7.
Step7: Denote the solutions obtained with different *ε*_*i*_ as *x*, the total transport time corresponding to solution *x* as *Z*_1_(*x*), the total transport cost as *Z*_2_(*x*), and the set of all feasible solutions as sol. Plot the total transport cost and total transport time of all feasible solutions, which is the approximation curve of the Pareto frontier in mathematical theory. The inflexion point of the Pareto frontier curve is found, and this solution is noted as the optimal dominated solution (called the MDP solution), which is the best compromise between the objective function *Z*_1_ and *Z*_2_. The solution satisfies:
MDP=A=(mintimeub−Z1(x))(mincostub−Z2(x))x∈solmax (14)

## 5 Case study

In this paper, the transport network conditions and historical material data of a large engineering project are selected to verify the feasibility of the model. The transport network consists of 82 nodes, including 10 suppliers with different supply capacities for goods, of which 9 supply points can be used as transport mode transit points. Additionally, there are 22 intermediate nodes in the network, including 3 transport mode transit points, and 50 work zones with varying demands for engineering materials. The transport network includes a total of 3 modes of transport: railway, road, and air transport.

The availability of suppliers in the network, the demand for goods in the work zones, the key processes at work zones, the cost and operating time of transit by any two transport modes at the transit points, the capacity of different transport modes of goods to pass through the nodes, the different maximum time limits for goods set by the work zones, the speed of transport by each transport mode, and the unit tariffs, distances, and capacity of the different modes of transport on the arcs of each segment are known.

**Fig 3:**
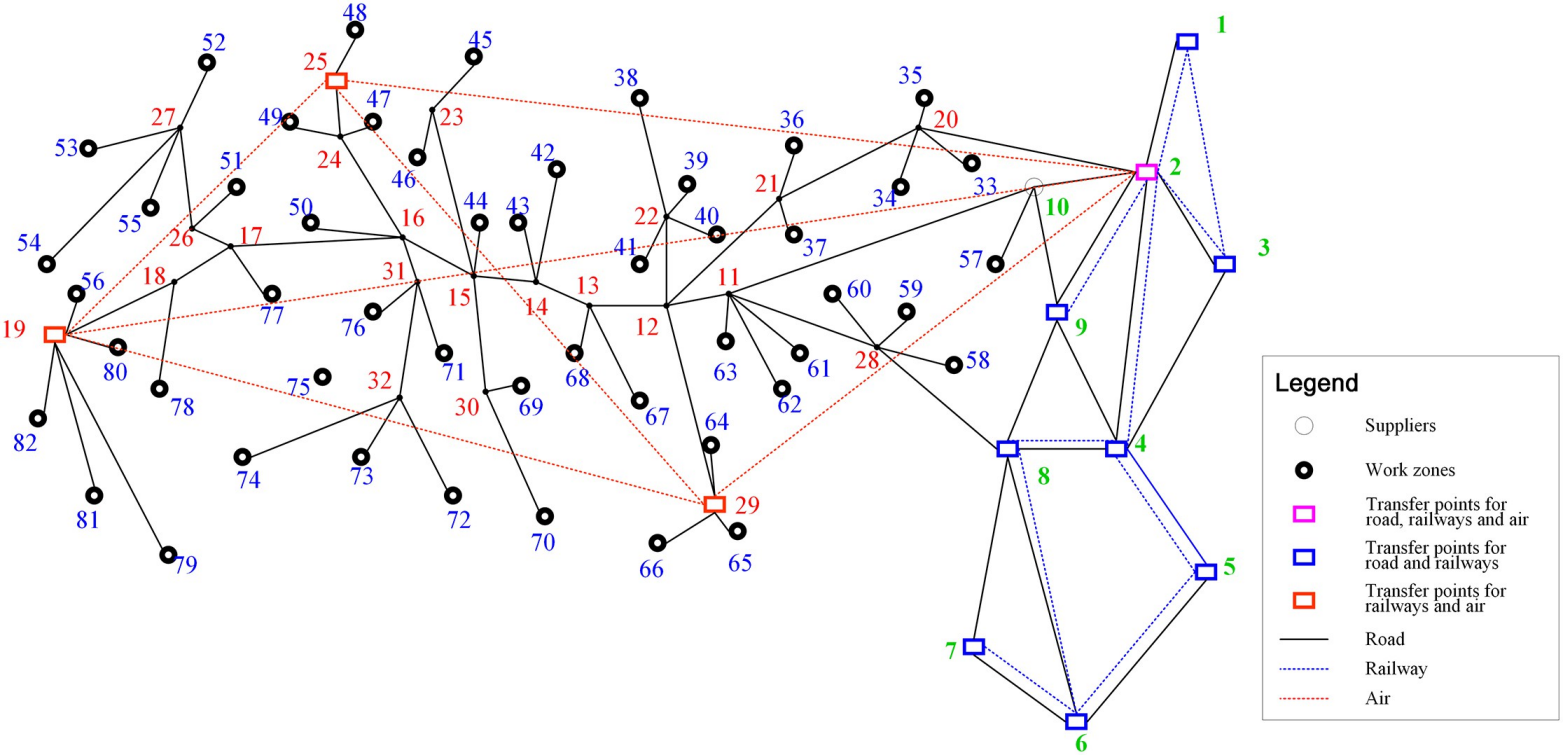
Network topology.

The topology of the cargo transport network is shown in Fig 3. Specifically, points 1–10 are suppliers, points 11–32 are intermediate nodes, and points 33–82 are work zones, each numbered with the construction point number corresponding to the transport task. Additionally, suppliers 1–9 can act as transport mode transit points for other transport tasks when they are not serving as supply points for a specific transport task. Nodes 19, 25 and 29 are considered ordinary transport mode transit points.

### 5.1 Results and analysis

**Fig 4:**
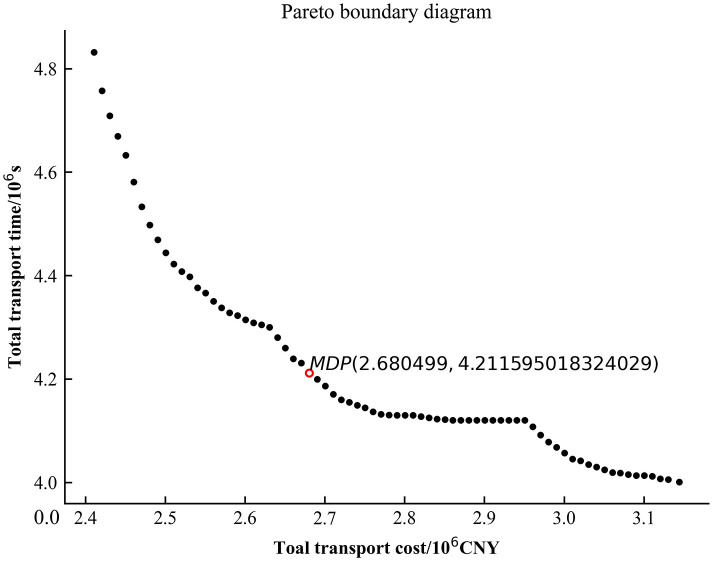
Pareto boundary diagram.

In order to efficiently solve the transportation task for 50 work zones, the problem was solved using Python programming language by invoking the Gurobi commercial solver with AMD R5-5600H, CPU (6 cores) 3.30GHz, and 16GB RAM. Setting the step *δ* of *ε*_*i*_ as 10000 CNY, *ε*_*i*+1_ = *ε*_*i*_ + *δ*. The model *P*_1_ is solved in several iterations to obtain a graph of the total transport time—total transport cost relationship (Fig 4 Pareto boundary diagram).

From Fig 4, it can be seen that the Pareto solution is uniformly distributed over the solution space range, with a stepwise downward trend in some intervals. The inflexion point of the Pareto frontier curve is found to obtain the MDP solution. The solved transport solution is shown in Table 2. The corresponding transport times and transport costs are shown in Table 3. Among them, there are three modes of transport, and each mode is converted at the transit point. The “Timeliness” indicates the proportion of the lead time for the delivery of materials to the construction site to the total time limit for material delivery, and a positive value means that the demand for materials at the construction site can be guaranteed on time. As can be seen from Table 3, the timeliness of the transport solution obtained according to the model solution is strong, and the materials can be transported to the work area in advance so that the guaranteed supply of engineering construction materials can be achieved.

**Table 2:** The solved transport solution.

Task	Transport origin	Transport route		
		Railway	Road	Air
33	1	1 -> 2	2 -> 20 -> 33	/
34	3	3 -> 2	2 -> 20 -> 34	/
35	5	5 -> 4 -> 2	2 -> 20 -> 35	/
36	4	4 -> 2 -> 20	20 -> 21 -> 36	/
37	6	6 -> 8	8 -> 9 -> 2 -> 20 -> 21 -> 37	/
38	2	/	2 -> 20 -> 21-> 12 -> 22 -> 38	/
39	2	/	2 -> 20 -> 21 -> 12 -> 22 -> 39	/
40	3	3 -> 2	2 -> 20 -> 21 -> 12 -> 22 -> 40	/
41	4	4 -> 8	8 -> 28 -> 11 -> 12 -> 22 -> 41	/
42	9	/	9 -> 10 -> 11 -> 12 -> 13 -> 14 -> 42	/
43	10	/	10 -> 11 -> 12 -> 13 -> 14 -> 43	/
44	1	1 -> 2	2 -> 20 -> 21 -> 12 -> 13 -> 14 -> 15 -> 44	/
45	8	/	8 -> 28 -> 11 -> 12 -> 13 -> 14 -> 15 -> 23 -> 45	/
46	8	/	8 -> 28 -> 11 -> 12 -> 13 -> 14 -> 15 -> 23 -> 46	/
47	9	9 -> 2	25 -> 24 -> 47	2 -> 25
48	1	1 -> 2	25 -> 48	2 -> 25
49	2	/	25 -> 24 -> 49	2 -> 25
50	3	3 -> 2	25 -> 24 -> 16 -> 50	2 -> 25
51	4	4 -> 8	8 -> 28 -> 11 -> 12 -> 13 -> 14 -> 15 -> 16 -> 17 -> 26 -> 51	/
52	10	/	10 -> 11 -> 12 -> 13 -> 14 -> 15 -> 16 -> 17 -> 26 -> 27 -> 52	/
53	1	1 -> 2	25 -> 24 -> 16 -> 17 -> 26 -> 27 -> 53	2 -> 25
54	3	3 -> 2	25 -> 24 -> 16 -> 17 -> 26 -> 27 -> 54	2 -> 25
55	5	5 -> 6 -> 7	7 -> 29; 19 -> 18 -> 17 -> 26 -> 27 -> 55	29 -> 19
56	8	/	8 -> 7 -> 29; 19 -> 56	29 -> 19
57	6	6 -> 8	8 -> 9 -> 10 -> 57	/
58	2	2 -> 9	9 -> 8 -> 28 -> 58	/
59	2	2 -> 9	9 -> 8 -> 28 -> 59	/
60	3	4 -> 8	3 -> 4; 8 -> 28 -> 60	/
61	4	4 -> 8	8 -> 28 -> 11 -> 61	/
62	9	/	9 -> 8 -> 28 -> 11 -> 62	/
63	10	/	10 -> 11 -> 63	/
64	1	1 -> 2 -> 9	9 -> 8 -> 7 -> 29 -> 64	/
65	8	/	8 -> 7 -> 29 -> 65	/
66	8	/	8 -> 7 -> 29 -> 66	/
67	9	/	9 -> 10 -> 11 -> 12 -> 13 -> 67	/
68	1	1 -> 2	2 -> 20 -> 21 -> 12 -> 13 -> 68	/
69	2	/	2 -> 20 -> 21 -> 12 -> 13 -> 14 -> 15 -> 30 -> 69	/
70	3	3 -> 2	25 -> 24 -> 16 -> 15 -> 30 -> 70	2 -> 25
71	4	4 -> 8	8 -> 28 -> 11 -> 12 -> 13 -> 14 -> 15 -> 16 -> 31 -> 71	/
72	4	4 -> 2	25 -> 24 -> 16 -> 31 -> 32 -> 72	2 -> 25
73	1	1 -> 2	25 -> 24 -> 16 -> 31 -> 32 -> 73	2 -> 25
74	3	3 -> 2	25 -> 24 -> 16 -> 31 -> 32 -> 74	2 -> 25
75	5	5 -> 6 -> 7	7 -> 29 -> 25 -> 24 -> 16 -> 31 -> 32 -> 75	/
76	8	/	8 -> 7 -> 29 -> 25 -> 24 -> 16 -> 31 -> 76	/
77	6	6 -> 7	7 -> 29 -> 19 -> 18 -> 17 -> 77	/
78	10	/	10 -> 11 -> 12 -> 29 -> 19 -> 18 -> 78	/
79	2	/	19 -> 79	2 -> 19
80	3	3 -> 2	19 -> 80	2 -> 19
81	4	4 -> 8	8 -> 7 -> 29; 19 -> 81	29 -> 19
82	9	/	9 -> 8 -> 7 -> 29; 19 -> 82	29 -> 19

**Table 3:** The calculation result.

Task	Transport origin	Total transport cost/CNY	Total transport time/s	Timeliness
33	1	144215.22	553786.45	64.39%
34	3	226535.10	652421.09	58.05%
35	5	423666.69	976128.06	37.23%
36	4	413538.41	952792.50	38.74%
37	6	597346.18	983061.14	36.79%
38	2	342778.72	987202.73	36.52%
39	2	329761.38	949712.78	38.93%
40	3	516467.14	1189940.28	23.49%
41	4	447299.07	858814.21	44.78%
42	9	247480.55	950325.33	38.89%
43	10	195025.32	748897.22	51.85%
44	1	445521.66	1283102.37	17.50%
45	8	410900.38	946714.47	39.13%
46	8	402352.77	927020.79	40.39%
47	9	1150515.25	637643.60	59.00%
48	1	583409.17	424442.64	72.71%
49	2	544833.29	313344.10	79.85%
50	3	862887.07	732316.03	52.91%
51	4	1163209.99	1329024.83	14.54%
52	10	293566.00	1127293.45	27.51%
53	1	586585.45	996712.34	35.91%
54	3	904263.84	1348504.09	13.29%
55	5	1186796.86	1478604.18	4.93%
56	8	701931.76	712912.44	54.16%
57	6	485684.76	940794.02	39.51%
58	2	240716.86	693264.55	55.42%
59	2	288615.83	831213.59	46.55%
60	3	379042.69	873314.35	43.85%
61	4	305324.98	586223.95	62.31%
62	9	168428.01	646763.57	58.41%
63	10	96074.53	368926.20	76.28%
64	1	446980.23	385490.50	75.21%
65	8	219965.52	506800.56	67.41%
66	8	237894.45	548108.82	64.76%
67	9	528742.61	870159.27	44.05%
68	1	361128.56	1106437.86	28.86%
69	2	723024.06	826533.52	46.85%
70	3	666232.78	1535000.33	1.30%
71	4	1164827.75	980693.50	36.94%
72	4	336857.16	1293531.49	16.83%
73	1	565785.87	916841.97	41.05%
74	3	810335.48	1077990.42	30.68%
75	5	958190.05	1497570.19	3.71%
76	8	379487.80	874339.88	43.78%
77	6	1123972.15	1011634.30	34.95%
78	10	879841.87	881893.63	43.29%
79	2	757173.16	528607.76	66.01%
80	3	986903.88	621775.59	60.02%
81	4	1057333.64	1125742.25	27.61%
82	9	486587.70	964158.41	38.00%

### 5.2 Sensitivity analysis

The model constructed in this paper considers the key processes at work zones, and in order to ensure faster delivery of construction materials to the site, the multi-objective model constructed in this paper is solved using the *ε* − constraint method, and the minimization of the total transport time is selected as the main objective for optimization. Firstly, in order to verify the effectiveness of introducing the key processes at work zones, we construct a model without considering the key processes at work zones on the basis of the original model, and use the same solution method as in this paper. Secondly, in order to verify the effectiveness of the *ε* − constraint method in solving the model constructed in this paper, we use the linear weighting method to solve the constructed model. Finally, in order to verify the effectiveness of minimizing the total transportation time as the main objective, we use the cost objective function as the primary objective and the time objective function as the sub-objective to solve the multi-objective model constructed in this paper using the *ε* − constraint method.

In this section, the above example is denoted as case1, and the following scenarios are set up for the sensitivity analysis of the model:

1. Considering the key processes at work zones, the multi-objective model constructed in this paper is solved using the *ε* − constraint method. (case1)
2. Without considering the key processes at work zones, remove the key processes in the work zone from the model constructed in this paper, and use the *ε* − constraint method to solve the problem. (case2)
3. Considering the key processes at work zones, the model constructed in this paper is solved using the linear weighting method, and the weights are both set to 0.5. (case3)
4. Considering the key processes at work zones, but the cost objective function is taken as the main objective, and the time objective function is taken as the sub-objective, using the *ε* − constraint method to solve this multi-objective model. (case4)

**Fig 5:**
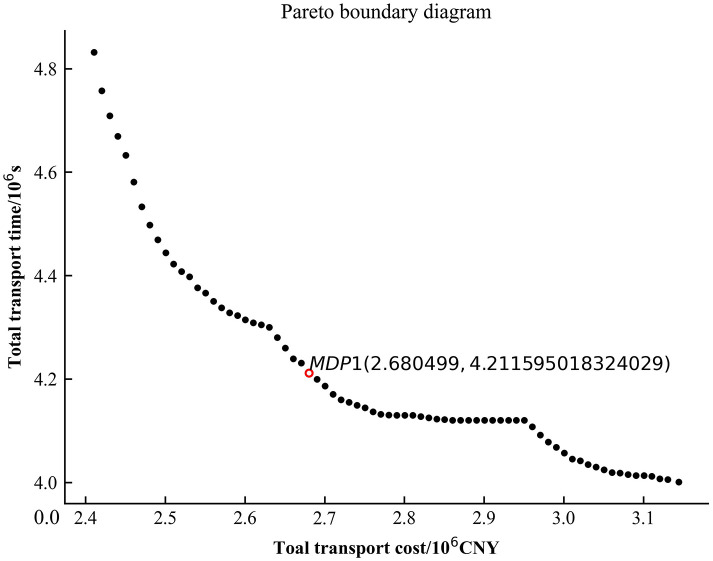
Pareto boundary diagram for case1.

**Fig 6:**
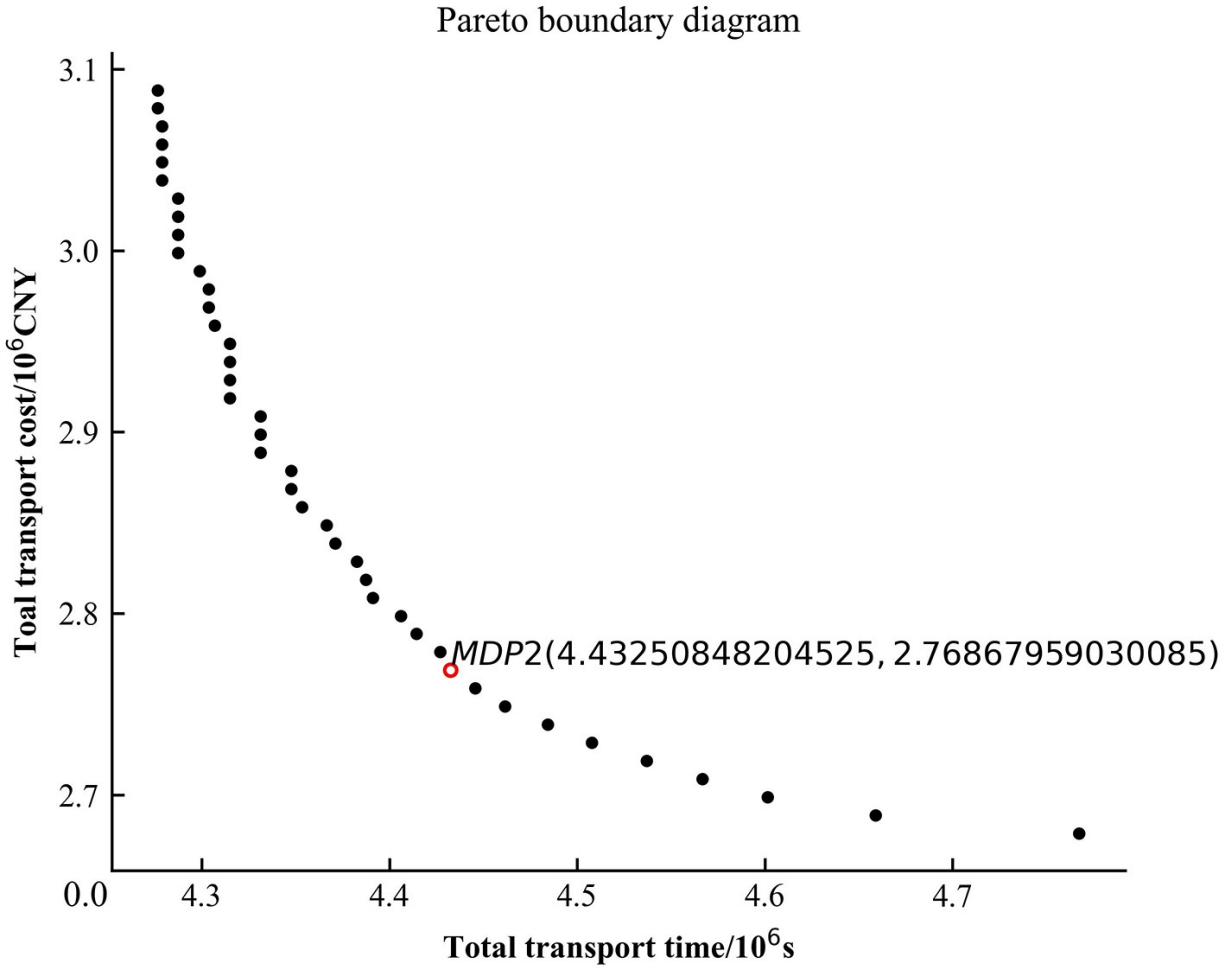
Pareto boundary diagram for case2.

**Fig 7:**
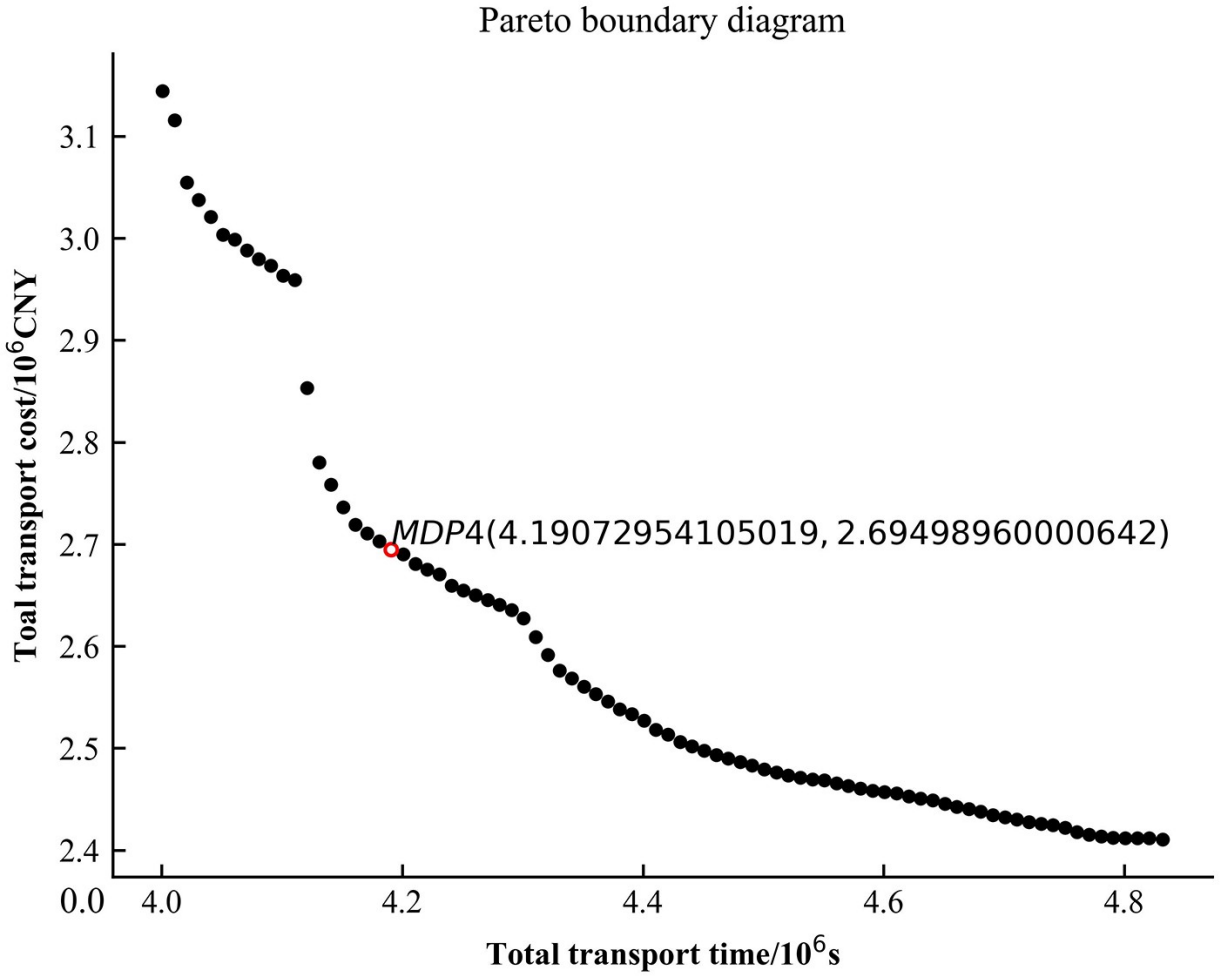
Pareto boundary diagram for case4.

**Fig 8:**
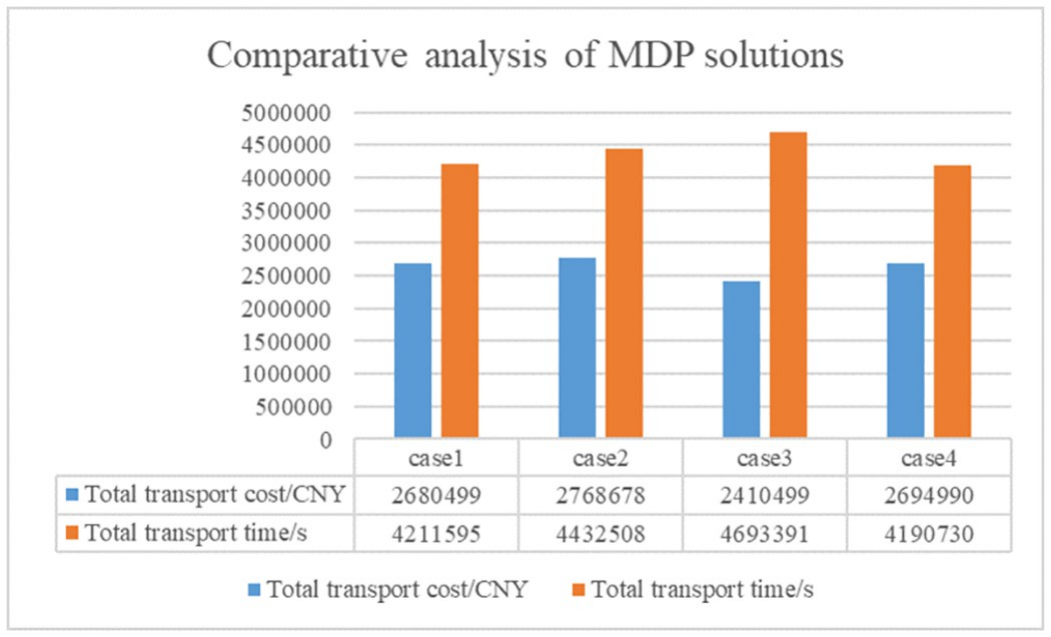
Comparative analysis of MDP solutions for case1-4.

Figs 5–7 are the plots of the total transport time—total transport cost relationship (Pareto boundary diagram) for case1, case2 and case4. Fig 8 is the comparative analysis of MDP solutions.

Analysis of Figs 5–7 reveals that the MDP solutions of different cases reflect the characteristics of their solution algorithms. Comparing the MDP solutions of case1 and case2, it can be found that the introduction of key processes at work zones can effectively reduce the total transport cost and total transport time. Comparing the MDP solutions of case1 and case4, it can be found that when using the *ε* − constraint method to solve the model, the selected main function has a greater impact on the solution results. Case1 reduces the total transport time by increasing a small amount of total transport cost; while case4 reduces the total transport cost by increasing a small amount of total transport time. Comparing the MDP solutions of case1, case2 and case4 reveals that the use of linear weighting alone sacrifices more of the total transport time and is less effective in solving the problem. The above cases validate the effectiveness of introducing key processes at work zones and the effectiveness of the *ε* − constraint method, respectively.

The transport time, transport cost and timeliness of each transportation task in cases1-4 are further outputted, and cases2, 3 and 4 are compared with case1. Where case2/case1 represents the change in transport time or transport cost or timeliness of case2 compared to case1 under the unified transportation task. Case3/case1 and case4/case1 are identical. Tables 4–6 shows the comparison of total transport time, total transport cost and timeliness for case1-4 respectively. From the tables, it can be found that the total time and total cost of transport obtained by solving the multi-objective optimization model using only the linear weighting method is relatively large and the timeliness is poor, which proves the effectiveness of the *ε* − constraint method in solving the multi-objective optimization model. In addition, the performance of case 2, which does not introduce the key processes at work zones, is relatively poor compared with the results of the other three cases that introduce key processes at work zones. This proves that the introduction of key processes at work zones is effective in solving the problem of planning the transportation route of engineering materials in large-scale engineering projects.

**Table 4:** Comparison of transport time.

Task	case1	case2	case3	case4	case2/case1	case3/case1	case4/case1
33–1	553786.45	553786.45	590668.44	553786.45	-2.10217E-16	0.066599672	0
34–3	652421.09	652421.09	707744.08	652421.09	0	0.084796452	0
35–5	976128.06	976128.06	1130115.46	976128.06	0	0.157753275	0
36–4	952792.50	952792.50	1054859.96	952792.50	0	0.107124542	0
37–6	983061.14	983061.14	1061669.84	983061.14	-1.18421E-16	0.079963187	0
38–2	987202.73	987202.73	987202.73	987202.73	0	0	0
39–2	949712.78	949712.78	949712.78	877960.82	-1.2258E-16	-1.2258E-16	-0.075551229
40–3	1189940.28	1189940.28	1245263.28	1189940.28	0	0.046492244	0
41–4	858814.21	858814.21	916486.17	858814.21	-1.35554E-16	0.067153005	0
42–9	950325.33	1005241.93	1005241.93	950325.33	0.05778716	0.05778716	0
43–10	748897.22	748897.22	748897.22	748897.22	0	0	0
44–1	1283102.37	839783.55	1319984.37	839783.55	-0.345505418	0.02874439	-0.345505418
45–8	946714.47	946714.47	946714.47	946714.47	1.22968E-16	1.22968E-16	0
46–8	927020.79	927020.79	1454441.38	927020.79	0	0.568941494	0
47–9	637643.60	637643.60	1130045.34	637643.60	0	0.772220931	0
48–1	424442.64	424442.64	461324.64	424442.64	0	0.086895123	0
49–2	313344.10	313344.10	313344.10	313344.10	0	0	0
50–3	732316.03	732316.03	1432475.54	732316.03	0	0.95608929	0
51–4	1329024.83	1329024.83	1292343.03	1234671.08	1.75189E-16	-0.02760054	-0.070994729
52–10	1127293.45	1127293.45	1127293.45	1127293.45	0	0	0
53–1	996712.34	996712.34	1033594.33	996712.34	-1.16799E-16	0.037003651	0
54–3	1348504.09	1348504.09	1403827.08	1395421.23	0	0.041025455	0.034791992
55–5	1478604.18	1478604.18	1530524.12	1478604.18	0	0.035114156	0
56–8	712912.44	712912.44	712912.44	712912.44	0	0	0
57–6	940794.02	862185.32	940794.02	862185.32	-0.083555699	0	-0.083555699
58–2	693264.55	773650.76	778893.11	693264.55	0.115953141	0.123514978	0
59–2	831213.59	750827.39	831213.59	831213.59	-0.096709442	0	0
60–3	873314.35	873314.35	930986.30	873314.35	0	0.066038025	0
61–4	586223.95	586223.95	643895.91	779342.94	0	0.098378708	0.329428678
62–9	646763.57	646763.57	646763.57	646763.57	0	0	0
63–10	368926.20	368926.20	368926.20	368926.20	-1.57776E-16	-1.57776E-16	0
64–1	385490.50	385490.50	1349603.62	385490.50	0	2.501003612	0
65–8	506800.56	506800.56	506800.56	506800.56	0	0	0
66–8	548108.82	548108.82	548108.82	548108.82	0	0	0
67–9	870159.27	870159.27	1284061.66	1320121.68	0	0.475662785	0.517103506
68–1	1106437.86	1069555.86	1106437.86	1069555.86	-0.033333996	0	-0.033333996
69–2	826533.52	1269852.35	1269852.35	826533.52	0.536359155	0.536359155	0
70–3	1535000.33	1535000.33	1147004.50	1535000.33	0	-0.252765958	0
71–4	980693.50	1096641.16	1082760.96	980693.50	0.118230276	0.104076819	0
72–4	1293531.49	1177583.83	1351203.45	1293531.49	-0.089636521	0.044584887	0
73–1	916841.97	916841.97	1526808.52	916841.97	-1.26974E-16	0.66529082	0
74–3	1077990.42	1077990.42	1133313.41	1077990.42	0	0.051320487	0
75–5	1497570.19	1497570.19	1534367.76	1497570.19	0	0.024571513	0
76–8	874339.88	874339.88	989790.73	874339.88	0	0.13204344	0
77–6	1011634.30	974836.73	1011634.30	974836.73	-0.036374375	1.15076E-16	-0.036374375
78–10	881893.63	881893.63	1116710.27	881893.63	0	0.266264124	0
79–2	528607.76	528607.76	528607.76	528607.76	-2.2023E-16	-2.2023E-16	0
80–3	621775.59	677098.58	677098.58	621775.59	0.088975821	0.088975821	0
81–4	1125742.25	1125742.25	1183414.20	1125742.25	0	0.05123016	0
82–9	964158.41	776764.29	964158.41	964158.41	-0.194360304	0	0

**Table 5:** Comparison of transport cost.

Task	case1	case2	case3	case4	case2/case1	case3/case1	case4/case1
33–1	144215.22	144215.22	136531.47	144215.22	2.01808E-16	-0.053279737	0
34–3	226535.10	226535.10	211167.60	226535.10	0	-0.067837161	0
35–5	423666.69	423666.69	370198.85	423666.69	0	-0.12620262	0
36–4	413538.41	413538.41	378098.32	413538.41	0	-0.085699634	0
37–6	597346.18	597346.18	559133.62	597346.18	0	-0.063970549	0
38–2	342778.72	342778.72	342778.72	342778.72	0	0	0
39–2	329761.38	329761.38	329761.38	304847.51	0	0	-0.075551
40–3	516467.14	516467.14	497257.76	516467.14	-1.12704E-16	-0.037193795	0
41–4	447299.07	447299.07	423269.09	447299.07	0	-0.053722404	0
42–9	247480.55	261781.75	261781.75	247480.55	0.05778716	0.05778716	0
43–10	195025.32	195025.32	195025.32	195025.32	-1.49231E-16	-1.49231E-16	0
44–1	445521.66	727624.77	435276.66	727624.77	0.633197296	-0.022995512	0.6331973
45–8	410900.38	410900.38	410900.38	410900.38	0	0	0
46–8	402352.77	402352.77	1023775.92	402352.77	-1.44668E-16	1.544473389	0
47–9	1150515.25	1150515.25	686659.49	1150515.25	0	-0.403172192	0
48–1	583409.17	583409.17	573164.17	583409.17	0	-0.017560572	0
49–2	544833.29	544833.29	544833.29	544833.29	0	0	0
50–3	862887.07	862887.07	578513.09	862887.07	0	-0.329561064	0
51–4	1163209.99	1163209.99	619027.87	643057.85	0	-0.467827927	-0.44717
52–10	293566.00	293566.00	293566.00	293566.00	0	0	0
53–1	586585.45	586585.45	578901.70	586585.45	0	-0.013099113	0
54–3	904263.84	904263.84	888896.34	1058150.06	0	-0.016994485	0.1701785
55–5	1186796.86	1186796.86	1168769.10	1186796.86	0	-0.015190263	0
56–8	701931.76	701931.76	701931.76	701931.76	0	0	0
57–6	485684.76	523897.33	485684.76	523897.33	0.078677707	0	0.0786777
58–2	240716.86	268628.73	216931.15	240716.86	0.115953141	-0.098811982	0
59–2	288615.83	260703.95	288615.83	288615.83	-0.096709442	0	0
60–3	379042.69	379042.69	359017.70	379042.69	-1.53565E-16	-0.05283042	0
61–4	305324.98	305324.98	281294.99	405907.78	0	-0.078702966	0.3294287
62–9	168428.01	168428.01	168428.01	168428.01	0	0	0
63–10	96074.53	96074.53	96074.53	96074.53	0	0	0
64–1	446980.23	446980.23	392043.27	446980.23	1.30224E-16	-0.122906909	0
65–8	219965.52	219965.52	219965.52	219965.52	-1.32311E-16	-1.32311E-16	0
66–8	237894.45	237894.45	237894.45	237894.45	4.15953E-15	4.15953E-15	0
67–9	528742.61	528742.61	1449647.76	802157.27	0	1.741688913	0.5171035
68–1	361128.56	371373.56	361128.56	371373.56	0.028369395	0	0.0283694
69–2	723024.06	440920.95	440920.95	723024.06	-0.390171121	-0.390171121	0
70–3	666232.78	666232.78	999652.30	666232.78	0	0.500454979	0
71–4	1164827.75	571167.27	1122299.64	1164827.75	-0.509655163	-0.036510212	0
72–4	336857.16	633687.40	324842.17	336857.16	0.881175386	-0.035667909	0
73–1	565785.87	565785.87	380317.95	565785.87	0	-0.327805856	0
74–3	810335.48	810335.48	794967.99	810335.48	-1.43663E-16	-0.018964365	0
75–5	958190.05	958190.05	945413.12	958190.05	0	-0.013334445	0
76–8	379487.80	379487.80	797710.53	379487.80	0	1.102071627	0
77–6	1123972.15	1141859.86	1123972.15	1141859.86	0.015914723	2.0715E-16	0.0159147
78–10	879841.87	879841.87	701752.99	879841.87	0	-0.202410096	0
79–2	757173.16	757173.16	757173.16	757173.16	1.5375E-16	1.5375E-16	0
80–3	986903.88	967694.50	967694.50	986903.88	-0.019464279	-0.019464279	0
81–4	1057333.64	1057333.64	1033303.66	1057333.64	0	-0.022726962	0
82–9	486587.70	632503.97	486587.70	486587.70	0.299876613	-1.19624E-16	0

**Table 6:** Comparison of timeliness.

Task	case1	case2	case3	case4	case2/case1	case3/case1	case4/case1
33–1	64.39	64.39	62.02	64.39	0	-0.036829935	0
34–3	58.05	58.05	54.49	58.05	-1.22404E-16	-0.061280777	0
35–5	37.23	37.23	27.33	37.23	0	-0.265921016	0
36–4	38.74	38.74	32.17	38.74	0	-0.169432585	0
37–6	36.79	36.79	31.73	36.79	0	-0.137394447	0
38–2	36.52	36.52	36.52	36.52	0	0	0
39–2	38.93	38.93	38.93	43.55	0	0	0.118502862
40–3	23.49	23.49	19.93	23.49	0	-0.151462071	0
41–4	44.78	44.78	41.07	44.78	0	-0.0828161	0
42–9	38.89	35.36	35.36	38.89	-0.09079005	-0.09079005	0
43–10	51.85	51.85	51.85	51.85	-1.3705E-16	-1.3705E-16	0
44–1	17.50	46.00	15.12	46.00	1.629263841	-0.135546922	1.629263841
45–8	39.13	39.13	39.13	39.13	0	0	0
46–8	40.39	40.39	6.48	40.39	0	-0.839602104	0
47–9	59.00	59.00	27.34	59.00	0	-0.536644654	0
48–1	72.71	72.71	70.34	72.71	0	-0.032617073	0
49–2	79.85	79.85	79.85	79.85	0	0	0
50–3	52.91	52.91	7.89	52.91	0	-0.850860557	0
51–4	14.54	14.54	16.90	20.61	0	0.162183161	0.417171169
52–10	27.51	27.51	27.51	27.51	0	0	0
53–1	35.91	35.91	33.54	35.91	0	-0.066039052	0
54–3	13.29	13.29	9.73	10.27	0	-0.267654025	-0.226986314
55–5	4.93	4.93	1.59	4.93	0	-0.677842979	0
56–8	54.16	54.16	54.16	54.16	0	0	0
57–6	39.51	44.56	39.51	44.56	0.127942605	0	0.127942605
58–2	55.42	50.25	49.92	55.42	-0.09326244	-0.099344512	0
59–2	46.55	51.72	46.55	46.55	0.111032751	0	0
60–3	43.85	43.85	40.14	43.85	0	-0.084577165	0
61–4	62.31	62.31	58.60	49.89	0	-0.059518453	-0.199302122
62–9	58.41	58.41	58.41	58.41	-1.21642E-16	-1.21642E-16	0
63–10	76.28	76.28	76.28	76.28	0	0	0
64–1	75.21	75.21	13.22	75.21	0	-0.824232961	0
65–8	67.41	67.41	67.41	67.41	0	0	0
66–8	64.76	64.76	64.76	64.76	0	0	0
67–9	44.05	44.05	17.43	15.12	0	-0.604201133	-0.65684038
68–1	28.86	31.23	28.86	31.23	0.082186067	0	0.082186067
69–2	46.85	18.35	18.35	46.85	-0.608397445	-0.608397445	0
70–3	1.30	1.30	26.25	1.30	0	19.2080306	0
71–4	36.94	29.49	30.38	36.94	-0.201821324	-0.177661105	0
72–4	16.83	24.28	13.12	16.83	0.443108971	-0.220400826	0
73–1	41.05	41.05	1.83	41.05	0	-0.955524206	0
74–3	30.68	30.68	27.13	30.68	-1.15781E-16	-0.115930181	0
75–5	3.71	3.71	1.34	3.71	0	-0.638516203	0
76–8	43.78	43.78	36.36	43.78	0	-0.169566174	0
77–6	34.95	37.32	34.95	37.32	0.067696628	0	0.067696628
78–10	43.29	43.29	28.20	43.29	0	-0.348751544	0
79–2	66.01	66.01	66.01	66.01	0	0	0
80–3	60.02	56.46	56.46	60.02	-0.059268852	-0.059268852	0
81–4	27.61	27.61	23.91	27.61	0	-0.134290171	0
82–9	38.00	50.05	38.00	38.00	0.317057421	0	0

## 6 Conclusions

In this paper, we introduce the key processes at work zones, take the total transport time and total transport cost as the optimization objectives, and take the node capacity, arc section capacity and time window limit as the constraints, a multi-objective route planning model for multimodal transport of engineering materials of large-scale engineering projects is constructed. The *ε* − constraint method is used to solve the multi-objective optimization model, and the feasibility of the model is verified by taking the data of the actual road network of a large-scale engineering project. Based on the multi-objective optimization model constructed in this paper, four different scenarios are designed and comparative analysis is carried out from two dimensions. In terms of whether to introduce the key process at work zones in the model, the comparison of the results of case2 and other cases shows that the introduction of the key process at work zones can effectively improve the timeliness of the delivery of engineering materials and reduce a certain amount of transportation costs. In terms of the solution effect of the *ε* − constraint method, the comparison of the results of case4 and other cassettes shows that the use of the *ε* − constraint method is very effective in solving the model; at the same time, the comparison of case1 and case4 shows that the constructors of large-scale engineering projects can select the main objective function according to the actual needs, use the *ε* − constraint t method to solve the model, and obtain a transportation route plan that is more in line with their needs.

There are several extensions of this work that could be considered for future research. As the number of work zones increases, the efficacy of utilizing the key processes at work zones may diminish. To address this issue, an enhanced computation method for the key processes at work zones can be devised. For instance, by designing a comprehensive evaluation system to acquire more comprehensive and reliable key processes at work zones. Additionally, to improve the efficiency and obtain desirable convergence and diversity of computational results, heuristic algorithms and multi-objective evolutionary computation methods can be designed and incorporated. Currently, advanced optimization algorithms (e.g., hybrid heuristics and metaheuristics, adaptive algorithms, self-adaptive algorithms, island algorithms, polyploid algorithms, hyper heuristics), have been well established in the fields of berth allocation and scheduling, multi-objective optimization, and transportation [36–40]. These approaches can optimize the model’s solution process by efficiently exploring the solution space and yielding high-quality results.

## Supporting information

S1 File Supporting information-data. (XLSX)
